# Acetaminophen-Induced Liver Injury Alters the Acyl Ethanolamine-Based Anti-Inflammatory Signaling System in Liver

**DOI:** 10.3389/fphar.2017.00705

**Published:** 2017-10-06

**Authors:** Patricia Rivera, Antoni Pastor, Sergio Arrabal, Juan Decara, Antonio Vargas, Laura Sánchez-Marín, Francisco J. Pavón, Antonia Serrano, Dolores Bautista, Anna Boronat, Rafael de la Torre, Elena Baixeras, M. Isabel Lucena, Fernando R. de Fonseca, Juan Suárez

**Affiliations:** ^1^Unidad de Gestión Clínica de Salud Mental, Instituto de Investigación Biomédica de Málaga, Hospital Regional Universitario de Málaga, Universidad de Málaga, Málaga, Spain; ^2^Department of Endocrinology, Fundación Investigación Biomédica del Hospital Infantil Universitario Niño Jesús, Madrid, Spain; ^3^Institut Hospital del Mar d'Investigacions Mèdiques, Barcelona, Spain; ^4^CIBER Fisiopatología Obesidad y Nutrición, Instituto Salud Carlos III, Madrid, Spain; ^5^Unidad de Gestión Clínica de Anatomía Patológica, Hospital Regional Universitario de Málaga, Málaga, Spain; ^6^Departamento de Especialidades Quirúrgicas, Bioquímica e Inmunología, Instituto de Investigación Biomédica de Málaga, Universidad de Málaga, Málaga, Spain; ^7^Servicio de Farmacología Clínica, Unidad de Gestión Clínica de Aparato Digestivo, Instituto de Investigación Biomédica de Málaga, Hospital Universitario Virgen de la Victoria, Universidad de Málaga, Málaga, Spain; ^8^Centro de Investigación Biomédica en Red de Enfermedades Hepáticas y Digestivas, Instituto Salud Carlos III, Madrid, Spain; ^9^Departamento de Biología Celular, Genética y Fisiología, Instituto de Investigación Biomédica de Málaga, Universidad de Málaga, Málaga, Spain

**Keywords:** paracetamol, PPARα, FAAH, OEA, toxicity, hepatic injury

## Abstract

Protective mechanisms against drug-induced liver injury are actively being searched to identify new therapeutic targets. Among them, the anti-inflammatory *N*-acyl ethanolamide (NAE)-peroxisome proliferators activated receptor alpha (PPARα) system has gained much interest after the identification of its protective role in steatohepatitis and liver fibrosis. An overdose of paracetamol (APAP), a commonly used analgesic/antipyretic drug, causes hepatotoxicity, and it is being used as a liver model. In the present study, we have analyzed the impact of APAP on the liver NAE-PPARα system. A dose-response (0.5–5–10–20 mM) and time-course (2–6–24 h) study in human HepG2 cells showed a biphasic response, with a decreased *PPAR*α expression after 6-h APAP incubation followed by a generalized increase of NAE-PPARα system-related components (*PPAR*α, *NAPE-PLD*, and *FAAH*), including the NAEs oleoyl ethanolamide (OEA) and docosahexaenoyl ethanolamide, after a 24-h exposure to APAP. These results were partially confirmed in a time-course study of mice exposed to an acute dose of APAP (750 mg/kg). The gene expression levels of *Ppar*α and *Faah* were decreased after 6 h of treatment and, after 24 h, the gene expression levels of *Nape-pld* and *Faah*, as well as the liver levels of OEA and palmitoyl ethanolamide, were increased. Repeated APAP administration (750 mg/kg/day) up to 4 days also decreased the expression levels of PPARα and FAAH, and increased the liver levels of NAEs. A resting period of 15 days completely restored these impairments. Liver immunohistochemistry in a well-characterized human case of APAP hepatotoxicity confirmed PPARα and FAAH decrements. Histopathological and hepatic damage (*Cyp2e1, Caspase3*, α*Sma, Tnf*α, and *Mcp1*)-related alterations observed after repeated APAP administration were aggravated in the liver of *Ppar*α-deficient mice. Our results demonstrate that the anti-inflammatory NAE-PPARα signaling system is implicated in liver toxicity after exposure to APAP overdose, and may contribute to its recovery through a long-term time-dependent response.

## Introduction

Drug-induced liver injury (DILI) refers to hepatotoxicity caused by xenobiotics, and it remains an important disease in clinical practice (Davern, 2012). Frequently, DILI is asymptomatic, so its recognition and diagnosis are often difficult, which cause it to be the most common cause of death from acute liver failure. Current hypotheses suggest that DILI pathogenesis is a multifactorial process involving a hepatotoxic potential of the drug, genetic factors and altered immunological responses (Grant and Rockey, 2012). Thus, there is a need for the identification of mechanisms contributing to DILI in order to develop protective/preventive therapeutic strategies. Some of these mechanisms are strictly related to dose, whereas others derive from individual susceptibility to the toxic effects of a certain drug. Following this clinical rationale, DILI is classified into intrinsic (dose-dependent) and idiosyncratic (unpredictable) types (Chalasani and Björnsson, 2010; Han et al., 2013). Acetaminophen, or paracetamol (N-acetyl-para-aminophenol, APAP), is the only drug whose hepatotoxicity is intimately related to the dose ingested (Andrade et al., 2009). Acetaminophen-induced liver injury (AILI) is currently the most frequent cause of DILI in humans, and human toxicity can be modeled in rodents when administered in an acute or cumulative overdose (Han et al., 2013; Li et al., 2013; Shah et al., 2013).

APAP is one of the most popular and most commonly used drugs with peroxide sensitive analgesic and antipyretic properties. The mechanism of action includes peripheral effects by cyclooxygenase (COX) inhibition and central effects by COX inhibition, descending serotoninergic pathways and increasing activity of the cannabinoid signaling system (ECS) (Jóźwiak-Bebenista and Nowak, 2014). APAP is metabolized in the liver primarily by CYP2e1 (Zaher et al., 1998), a cytochrome P450 isoform, producing N-acetyl-p-benzoquinone imine (NAPQI), which is normally detoxified by conjugation with reduced glutathione (GSH) when used at therapeutic doses. However, GSH is exhausted when an acute or repeated APAP overdose is administered; excessive NAPQI binds covalently to cellular proteins, inducing mitochondrial dysfunction, lipid peroxidation, oxidative stress and DNA fragmentation (Gul et al., 2012; Li et al., 2013). APAP is also metabolized through a two-step process in the rodent central nervous system (spinal cord and brain) (Kelley and Thayer, 2004; Högestatt et al., 2005; Ottani et al., 2006). APAP is subject to deacetylation to p-aminophenol that in turn reacts with arachidonic acid through the fatty acid amide hydrolase (FAAH)—*N*-acyl ethanolamine (NAE) degradation enzyme. This reaction results in the synthesis of the active metabolite fatty acid amide N-(4-hydroxyphenyl) arachidonylamide (AM404), a well-characterized indirect activator of the cannabinoid CB1 receptors, as well as a direct ligand of the transient receptor potential vanilloid-1 (TRPV1). AM404 is able to block the reuptake of the NAE anandamide (AEA) in neurons, which, in turn, produces an increased level of AEA, activating the CB1 receptor and mediating antinociceptive actions (Beltramo et al., 1997; Ayoub et al., 2011).

The peroxisome proliferator-activated receptor alpha (PPARα) is a member of PPAR nuclear receptors that have emerged as a key player in controlling metabolic homeostasis, inflammation and fibrogenesis (Charbonnel, 2009; Gross et al., 2016). PPARα is predominantly present in the liver and is known to regulate the transcription of a wide variety of peroxisomal, microsomal, mitochondrial, and cytosolic proteins such as acyl coenzyme A oxidase, peroxisomal bifunctional enzyme, cytochrome P450 CYP4A, and liver fatty acid-binding protein. PPARα controls the lipid flux in the liver and its activation inhibits inflammatory genes induced by nuclear factor-κB. PPARα activation can confer liver protection in disease models of non-alcoholic steatohepatitis (NASH), alcohol liver disease (ALD) and liver fibrosis. Thus, fibrates and derivatives acting on activating PPARα interfere with NASH pathogenesis by reducing steatosis, inflammation and fibrosis (Ratziu et al., 2016), and by enhancing mitochondrial functions (Li et al., 2014). Moreover, PPARα may play a key role in the resistance to APAP hepatotoxicity (Mehendale, 2000). Pre-exposure to peroxisome proliferators afforded remarkable protection from APAP-induced liver injury assessed by decreased arylation of cytosolic proteins, hepatic GSH depletion and necrosis (Chen et al., 2009).

In recent years, a group of lipids related to the acylethanolamides (NAEs) has been identified as endogenous ligands for PPARα. Thus, the PPARα signaling system consists of this receptor and high-affinity NAEs that activate PPARα, such as oleoylethanolamide (OEA) and palmitoilethanolamide (PEA) (Fidaleo et al., 2014). It also includes the machinery dedicated to NAE biosynthesis and release, such as N-acyl phosphatidylethanolamide-specific phospholipase D (NAPE-PLD), as well as mechanisms for cellular uptake and hydrolysis, such as fatty acid amide hydrolase (FAAH) and N-acylethanolamide-hydrolyzing acid amidase (NAAA) (Ueda et al., 2010). Recent reports indicate that OEA reduced fibrosis development through inhibition of the pro-fibrotic cytokine TGF-β1-induced hepatic stellate cell activation (Chen et al., 2015). Hepatocytes highly express the NAE-degrading enzyme FAAH, which protects them from AEA-induced injury and cell death (Siegmund et al., 2013). Thus, hepatocyte cell death is considered to promote fibrogenesis, whereas elimination of activated hepatic stellate cells could represent a cannabinoid-independent mechanism to attenuate the fibrogenic response (Bataller and Brenner, 2005).

Although PPARα may be a potential therapeutic target in APAP-induced hepatotoxicity, the involvement of the liver machinery dedicated to the signaling, synthesis and degradation of the endogenous fatty acid derivatives NAEs has not been fully investigated. The present study is designed with *in vitro* and *in vivo* experimental approaches to investigate how the hepatic endogenous NAE-PPARα signaling system is affected across the different stages of APAP-induced liver injury. Studies involved a measurement of NAE levels and the liver gene/protein expressions of PPARα and enzymes that synthesize (NAPE-PLD) and degrade (FAAH) NAEs. This study was first performed in a human liver HepG2 cell line cultured with APAP in a dose-response (0.5, 5, 10, and 20 mM) and time-course (2, 6, and 24 h) manner. Then, experiments were carried out in several mouse models of APAP-induced liver injury (750 mg/kg/day) in order to analyse the effects of 1) acute APAP in a time-course (6, 24, and 48 h) manner, 2) repeated APAP administration for 3 and 4 days, and 3) a resting period of 6 and 15 days after a 4-day repeated APAP administration. Immunohistochemical analysis was also performed in the mouse liver after APAP administration and in the liver of a human patient with acute hepatic failure caused by APAP overdose. Finally, hepatic damage was compared to that in the liver of *Ppar*α-deficient mice. Our results indicate that acute and repeated APAP overdose-induced liver injury alters the liver expression of the NAE-PPARα signaling-related machinery in a time-response manner.

## Materials and methods

### Ethics statement

The protocols for animal care and use were approved by the Ethics and Research Committee at the University Regional Hospital of Malaga and University of Malaga (Ref. no. 24-2015-A). All experimental animal procedures were carried out in strict accordance with the European Communities directive 86/609/ECC (24 November 1986) and Spanish legislation (BOE 252/34367-91, 2005) regulating animal research. All efforts were made to minimize animal suffering and to reduce the number of animals used.

Liver samples (biopsies) were obtained after written informed consent from all the patients and donor's family, as requested by specific legal and clinical guidelines of the Ethics and Research Committee at the University Hospitals of Malaga. Research procedures were approved and supervised by the Ethics and Research Committee at the University Hospitals of Malaga (Ref. no. PI-0337-2012) and were conducted according to the principles expressed in the Declaration of Helsinki.

### Human HepG2 cell culture and *in vitro* treatment with APAP

The human hepatoblastoma cell line HepG2 was cultured in Minimum Essential Medium (MEM; 5 mM glucose) supplemented with 1% L-glutamine, 10% heat-inactivated fetal bovine serum (FBS), 100 U/mL penicillin and 100 μg/mL streptomycin (37°C in humidified atmosphere of 95% air and 5% CO_2_). HepG2 cells (1.5 × 10^4^ cells/well) were cultured in MEM containing APAP (0.5, 5, 10, or 20 mM) or vehicle (0.5% DMSO) for 2, 6, and 24 h (*n* = 3). Then, culture media and HepG2 cells were collected and snap-frozen for LC/MS-MS and molecular analyses, respectively.

### Animal model

Crl:CD1 (ICR) male mice (Charles Rivers Laboratories, Barcelona, Spain) and *Ppar*α knockout (KO) male mice with C57Bl/6 background (The Jackson Laboratories, Bar Harbor, ME, USA), weighting 25–30 g and aging 3–4 months old, were housed in standard conditions (vivarium of the University of Malaga) at 20 ± 2°C room temperature, 40 ± 5% relative humidity and a 12-h light/dark cycle with dawn/dusk effect. Water and standard rodent chow (Prolab RMH 2500, 2.9 kcal/g) were available *ad libitum*. The animals were handled for 10 min daily and habituated to an oral gavage procedure for 1 week prior to experimentation in order to minimize stress effects.

### *In vivo* treatment with APAP

APAP (Acetaminophen, cat. no. A7085, Sigma-Aldrich, St. Louis, MO, USA) was orally (gavage) administered (750 mg/kg/day; 10 mL/kg), as was previously described (Eakins et al., 2015). We generated four experimental groups (*n* = 6/group) of CD1 mice: A single vehicle administration group (vehicle group); and one APAP administration and sacrificed 6 h later (APAPx1 6 h group), 24 h later (APAPx1 24 h group) or 48 h later (APAPx1 48 h group) groups. We also generated three experimental groups (*n* = 6/group) of CD1 mice: A repeated vehicle administration for 4 days group (vehicle group); a repeated APAP administration for 3 days group (APAPx3 group); and a repeated APAP administration for 4 days group (APAPx4 group). For analysing the putative recovery effect due to the cessation of the repeated APAP administration, we generated two additional experimental groups (*n* = 6/group) of CD1 mice: A repeated APAP administration for 4 days and sacrificed 6 days later group (APAPx4 6d group); and a repeated APAP administration for 4 days and sacrificed 15 days later group (APAPx4 15d group). Finally, *Ppar*α KO mice were also exposed to a repeated vehicle or APAP administration for 3 days (*n* = 6/group). Animals were fasted for 12 h before sacrifice to avoid food-induced changes in liver genes (Vida et al., 2015; Gavito et al., 2016).

### Sample collection

Animals were intraperitoneally anesthetized (sodium pentobarbital, 50 mg/kg) and blood samples were transcardially collected (K3-EDTA tubes) and centrifuged (1,600 *g*, 10 min, 4°C). The plasma and liver portions were snap-frozen in liquid nitrogen and stored at −80°C for biochemical, molecular and LC/MS-MS analyses. Other batch of animals was transcardially fixed with 4% formaldehyde in 0.1 M phosphate buffer. Portions of liver were collected for histological and immunohistochemical analysis.

### Biochemical analysis

Total cholesterol (TChol), high-density lipoprotein (HDL)-cholesterol, triglycerides (TG), phosphatase alkaline (ALP) and the hepatic enzymes alanine aminotransferase (ALT), aspartate aminotransferase (AST) and gamma-glutamyl transpeptidase (γGT) were analyzed using commercial kits according to the manufacturer's instructions in a Hitachi 737 Automatic Analyzer (Hitachi Ltd., Tokyo, Japan) as were previously described (Warnick et al., 1990; Crespillo et al., 2011).

### Quantification of endocannabinoids and related compounds

The determination of the NAEs N-arachidonoyl ethanolamide (AEA or anandamide), N-docosahexaenoyl ethanolamide (DHEA), N-oleoyl ethanolamide (OEA) and N-palmitoyl ethanolamide (PEA) in human HepG2 culture cell media and mouse liver samples was based on methodology previously described (Pastor et al., 2014), and it was adapted for the extraction of endocannabinoids from liver tissue. Twenty microlitres were injected into the LC/MS-MS system.

### RNA isolation and RT-qPCR analysis

We performed real-time qPCR (TaqMan, ThermoFisher Scientific, Waltham, MA, USA) in human HepG2 cells and mouse liver as described previously (Suarez et al., 2014). Real-time qPCR was performed using a CFX96 Touch™ Real-Time PCR Detection System (Bio-Rad). Primers used were obtained based on TaqMan® Gene Expression Assays and the FAM™ dye label format (ThermoFisher) (Table 1). Absolute values from each sample were normalized with regard to the housekeeping gene *GAPDH* (human HepG2 cells) or β*2-microglubulin* (mouse liver). The relative quantification was calculated using the ΔΔCt method and normalized to the control group.

**Table 1:** Primer references for TaqMan® gene expression assays (ThermoFisher).

**Gene ID**	**GenBank accession n°**	**Assay ID**	**Amplicon length**
*GAPDH*	NM_001256799.1	Hs02758991_g1	93
*β2-microglobulin (B2m)*	NM_009735.3	Mm00437762_m1	77
*Cyp2e1*	NM_021282.2	Mm00491127_m1	83
*αSma (Acta2)*	NM_007392.3	Mm00725412_s1	95
*Caspase3*	NM_001284409.1	Mm01195085_m1	70
*Tnfα*	NM_001278601.1	Mm00443258_m1	81
*Il6*	NM_031168.1	Mm00446190_m1	78
*Mcp1 (Ccl2)*	NM_011333.3	Mm00441242_m1	74
*PPARα*	NM_001001928.2	Hs00947536_m1	62
*Pparα*	NM_001113418.1	Mm00440939_m1	74
*NAPEPLD*	NM_001122838.1	Hs00419593_m1	79
*Napepld*	NM_178728.5	Mm00724596_m1	85
*FAAH*	NM_001441.2	Hs01038660_m1	122
*Faah*	NM_010173.4	Mm00515684_m1	62

### Western blot analysis

Protein levels from mouse liver were measured as previously described (Crespillo et al., 2011). An appropriate combination of primary and HRP-conjugated secondary antibodies was used (Table 2). Western blots showed that each primary antibody detected a protein of the expected molecular size. The protein intensity was quantified with the image processing software ImageJ (Rasband, W.S., ImageJ, U.S., NIH, http://imagej.nih.gov/ij, 1997–2012). The results were expressed as the protein/β-actin ratio.

**Table 2:** Antibodies used for protein expression by western blotting.

**Antigen**	**Immunogen**	**Manufacturing details**	**Dilution**
βactin	A modified synthetic β-cytoplasmic actin N-terminal peptide	SIGMA. Mouse monoclonal antibody. Cat. n°: A5316	1:1000
PPARα	Synthetic peptide (VDTESPICPLSPLEADD)	Fitzgerald. Rabbit polyclonal antibody. Cat. n°: 20R-PR021	1:300
NAPE-PLD	A 13-aa peptide comprising part of both the C-terminal and the N-terminal regions of NAPE-PLD (MDENSCDKAFEET)	Developed by our group. Rabbit polyclonal antibody	1:100
FAAH	Synthetic peptide from rat FAAH, aa 561-579 (CLRFMREVEQLMTPQKQPS)	Cayman. Rabbit polyclonal antibody. Cat. N°: 101600	1:200

### Human subjects

Human liver biopsies were retrospectively selected from 27 patients with DILI and from 8 brain-dead, heart-beating, non-diabetic, non-obese adult matched controls, all diagnosed by clinical, biochemical, and histopathological criteria. Liver samples were retrieved from the tissue bank of Pathology Service at the University Regional Hospital and University Virgen de la Victoria Hospital of Malaga. The control group consisted of liver samples selected from healthy donors. We histopathologically confirmed the absence of macroscopic and microscopic alterations. Regarding the etiology of the DILI patients analyzed, we could only select a unique case of acute hepatic failure, due to an abusive consumption of paracetamol over time, without other relevant comorbidities. At the moment of emergency admission, the patient (male, 40 years old, several suicide attempts) showed low blood pressure (70/40), drowsiness, peripheral cyanosis, conjunctival icterus, right upper quadrant abdominal pain, and altered circulating levels of aspartate aminotransferase (AST > 4,600 U/L), creatinine (4.6 mg/dL), leukocytosis (39,000,000 U/L), among others. The APAP concentration in serum measured at admission of the patient to the emergency room (over 4 h post ingestion) was 220 μg/mL, indicating possible liver toxicity (Rumack-Matthiew monogram). The clinicians proceeded with N-acetylcysteine treatment. The post-mortem analysis of the liver (2 days after emergency admission) indicated extensive necrosis, congestion, infiltration, hemorrhage and ischaemia, all signs compatible with a hepatotoxic etiology.

### Histology, immunohistochemistry, and immunofluorescence

Sections carried out at centrolobulillar planes from mouse and human liver samples were stained with haematoxylin-eosin (H-E) for histological assessment of tissue injury. Adjacent sections were incubated in the following diluted primary antibody for 48 h at 4°C: rabbit anti-PPARα (1:50, Fitzgerald, cat. no. 20R-PR021), rabbit anti-MAGL (1:50, Cayman, cat. no. 100035), rabbit anti-FAAH (1:50, Cayman, cat. no.101600), rabbit anti-DAGLβ (1:25, developed by our group), and rat monoclonal anti- B220 or CD45R (clone RA3-6B2) (1:50, ThermoFisher, cat. no. RM2601). For immunohistochemistry, sections were first incubated in a 1:500 dilution of a biotin-conjugated donkey anti-rabbit secondary antibody (Amersham) for 1 h, and then incubated in the dark for 1 h in a 1:2000 dilution of ExtrAvidin peroxidase (Sigma). Immunolabeling was revealed by exposing to 0.05% diaminobenzidine (DAB; Sigma), 0.05% nickel ammonium sulfate, and 0.03% H_2_O_2_ in 0.1 M phosphate-buffered saline (pH 7.4). For immunofluorescence, sections were incubated in a donkey anti-rat IgG (H+L) labeled with AlexaFluor® 488 (1:500, MolecularProbes, Invitrogen, Paisley, UK, cat. no. A21208) for 2 h. For Annexin V detection, we used the FITC Annexin V apoptosis detection kit I according to the manufacturer's instructions (BD Biosciences, Madrid, Spain, cat. no. 556547). Digital high-resolution microphotographs were taken with an Olympus BX41 microscope equipped with an Olympus DP70 digital camera (Olympus Europa GmbH, Hamburg, Germany).

### Statistical analysis

All data are represented as the mean ± S.E.M. (standard error of the mean) of at least six determinations per experimental group (*n* = 6). Kolmogorov-Smirnov normality tests indicated that all data followed a Gaussian distribution (*P* > 0.1), so we selected a parametric statistical test. Statistical analysis was performed using one- or two-way ANOVA following Bonferroni's *post hoc* test for multiple comparisons or Student's *t*-test when appropriate. Correlation analyses were also performed through the Pearson correlation coefficient (*r*). *P* < 0.05 was considered to be significant.

## Results

### APAP effect on the NAE-PPARα signaling system in human HepG2 cells in a dose and time-dependent manner

**Figure 1:**
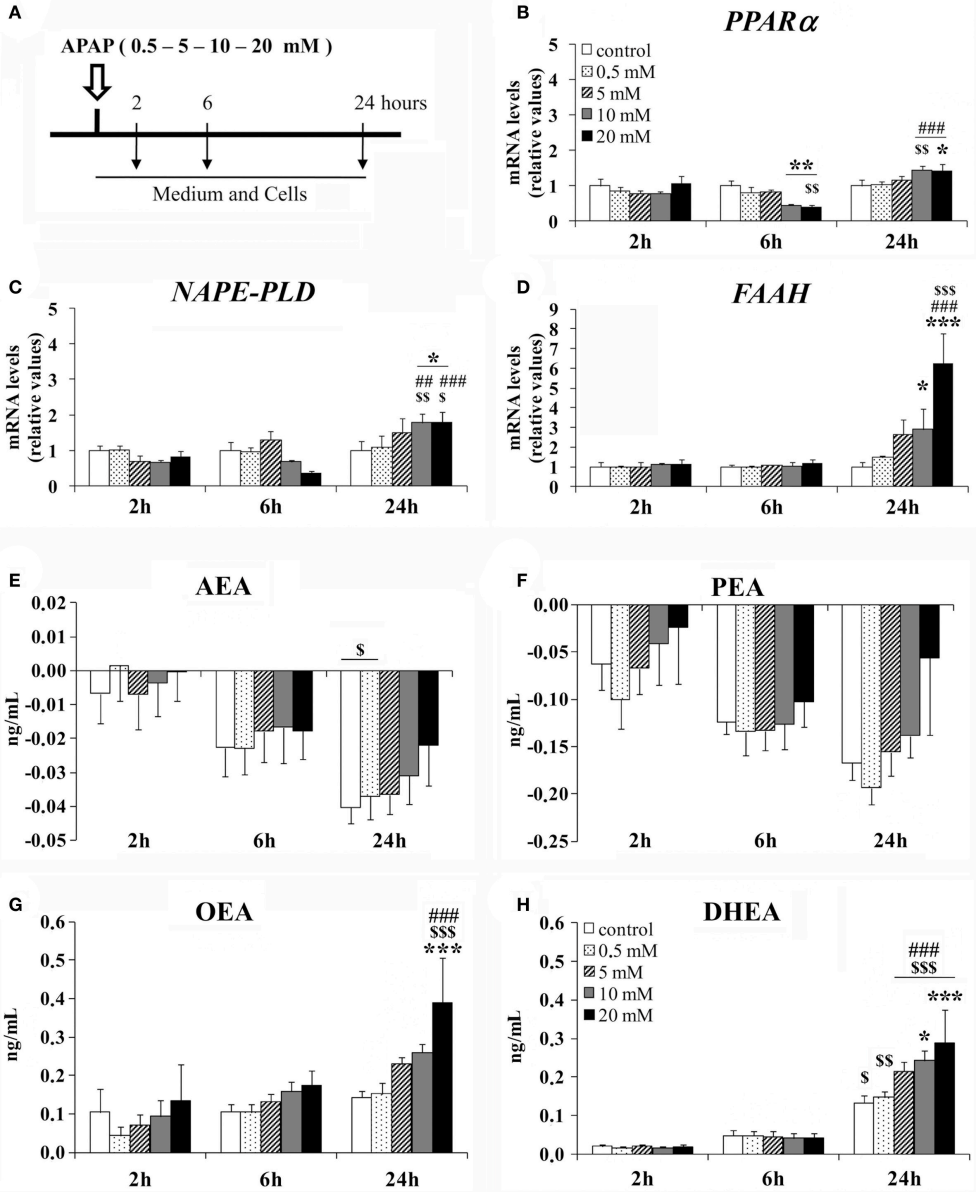
Effects of APAP incubation at doses of 0.5, 5, 10, and 20 mM for 2, 6, and 24 h on the gene expression levels of NAE/PPARα-related components in a human hepatoblastoma cell HepG2 line. **(A)** Timetable of the experimental design. **(B–D)** Quantification of the mRNA levels of *PPAR*α, *NAPE-PLD*, and *FAAH* in HepG2 cells. **(E–H)** Quantification of the concentration of AEA, PEA, OEA, and DHEA in HepG2 cell media. Quantification of the concentration of AEA, PEA, OEA, and DHEA in HepG2 cell media. Histograms represent the mean ± S.E.M. (*n* = 3). Bonferroni test: ^*/**/***^P < 0.05/0.01/0.001 vs. the respective timing group treated with vehicle; ^$/$$/$$$^*P* < 0.05/0.01/0.001 vs. the respective dosing group treated for 2 h; ^##/###^*P* < 0.01/0.001 vs. the respective dosing group treated for 6 h.

Human hepatic HepG2 cells were incubated with APAP at different doses (0.5, 5, 10, and 20 mM) for 2, 6, and 24 h (Figure 1A). Two-way ANOVA showed a time effect on the gene expression of *PPAR*α, *NAPE-PLD*, and *FAAH* [*F*_(2, 75)_ > 10.00, *P* < 0.001]. A dose effect on the gene expression of *FAAH* was specifically found [*F*_(4, 75)_ = 5.53, *P* < 0.001]. Interaction between dose and time was observed in the gene expression of *PPAR*α [*F*_(8, 75)_ = 3.90, *P* < 0.001], *NAPE-PLD* [*F*_(8, 75)_ = 2.51, *P* < 0.05] and *FAAH* [*F*_(8, 75)_ = 4.51, *P* < 0.001] (Figures 1B–D). Thus, increased doses of APAP differentially affected the HepG2 cell gene expression of the NAE-PPARα signaling machinery in an incubation time-dependent manner. Bonferroni analysis indicated that HepG2 cells cultured with 10 and 20 mM of APAP for 6 h exhibited a decrease in the gene expression of *PPAR*α (^**^P < 0.01), but it was increased after 24 h of APAP incubation compared to the respective controls (^*^P < 0.05) (Figure 1B). Gene expression levels of *NAPE-PLD* and *FAAH* were also increased in HepG2 cells cultured with 10 and 20 mM of APAP for 24 h (^*/***^P < 0.05/0.001) (Figures 1C,D). HepG2 cells did not exhibit any change at any concentration in the gene expression of *PPAR*α after 2 h of APAP incubation (Figure 1B), and in the gene expression levels of *NAPE-PLD* and *FAAH* after 2 and 6 h of APAP incubation (Figures 1C,D).

After LC/MS-MS analysis, two-way ANOVA indicated a time effect on the medium concentration of NAEs in a dose-independent manner [AEA: *F*_(2, 75)_ = 14.41, *P* < 0.001; PEA: *F*_(2, 75)_ = 7.56, *P* < 0.01; OEA: *F*_(2, 75)_ = 12.83, *P* < 0.001; DHEA: *F*_(2, 75)_ = 78.12, *P* < 0.001]. The commercial medium contains acylethanolamides, and we obtained a decrease in the concentrations of AEA and PEA, probably reflecting a negative balance between production and degradation of these NAEs. Contrariwise, we observed an increase (production) in the concentration of OEA and DHEA contents over time in the culture media after cell incubation relative to the basal values of NAE contents in the culture media (MEM) before cell incubation (Figures 1E–H). Interestingly, a dose effect on the medium concentration of OEA [*F*_(4, 75)_ = 4.29, *P* < 0.01] and an interaction between factors (time and dose) in the medium content of DHEA [*F*_(8, 75)_ = 2.27, *P* < 0.05] were specifically observed. Thus, we observed an increased content of OEA and DHEA in the medium of the HepG2 cells cultured with 20 mM of APAP for 24 h compared with the respective controls (^***^P < 0.001; Figures 1G,H). HepG2 cells did not exhibit any change at any concentration in the medium content of AEA and PEA when cells were incubated with APAP for 2, 6, and 24 h (Figures 1E,F).

### Effect of acute APAP on hepatic injury-related factors and the NAE-PPARα signaling system in the mouse liver in a time-dependent manner

**Figure 2:**
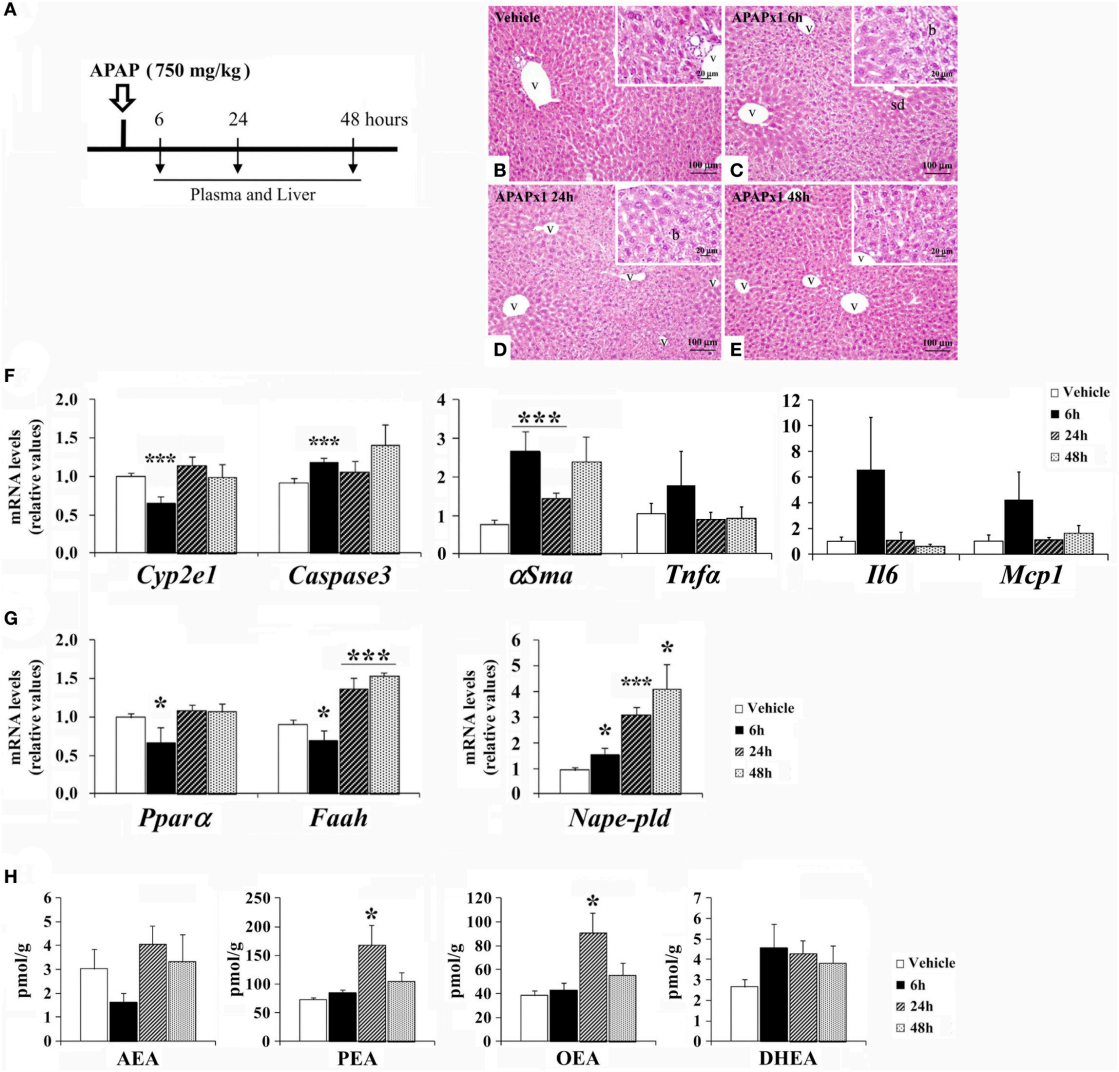
Effects of acute oral overdose of APAP (750 mg/kg) on histopathology, the gene expression levels of hepatic injury-related factors and NAE/PPARα-related components, and the NAE levels in the mouse liver after 6, 24, and 48 h from treatment. **(A)** Timeline of the experimental design. **(B–E)** Representative photomicrographs showing low and high (insets)—magnification views of H-E-stained liver sections: Vehicle group, APAPx1 6 h group, APAPx1 24 h group, and APAPx1 48 h group. b, ballooning; sd, sinusoidal dilatation; v, blood vessel. **(F,G)** Quantification of the mRNA levels of *Cyp2e1, Caspase3*, α*Sma, Tnfa, Il6, Mcp1, Ppar*α, *Nape-pld*, and *Faah*. **(H)** Quantification of the liver levels of AEA, PEA, OEA, and DHEA. Histograms represent the mean ± S.E.M. (*n* = 6). Bonferroni test, ^*/***^P < 0.05/0.001 vs. vehicle-treated group.

Mice were treated with an acute oral overdose of APAP (750 mg/kg) and sacrificed 6, 24, and 48 h later (Figure 2A). We observed a time-response effect of acute APAP on the histopathology in mouse liver (Figures 2B–E). Acute APAP-induced liver injury was addressed by mild sinusoidal dilatation, inflammation (lymphocytes infiltration) and ballooning in the hepatic parenchyma. The liver of vehicle group exhibited a hepatic parenchyma surrounding the central vein (Figure 2B). Moderate sinusoidal dilatation, minimal lymphocyte infiltration and ballooning in hepatic parenchyma were found after 6 and 24 h of acute APAP administration (Figures 2C,D). No necrosis was detected. The hepatic parenchyma alterations were decreased 48 h later, becoming a hepatic histology similar to the vehicle group (Figure 2E). Histopathology was associated with changes in the expression of hepatic damage-related factors (Figure 2F). Our results indicated a decreased gene expression of the APAP-related damage marker *Cyp2e1* and an increased gene expression of the cell proapoptosis marker *Caspase3* and the hepatic fibrosis marker α*Sma* in the liver of mice sacrificed 6 h after treatment (^***^P < 0.001). The liver of these mice also exhibited a non-significant increase in the gene expression levels of the inflammatory cytokines *Tnf*α, *Il6* and *Mcp1*. After 48 h of treatment, the gene expression levels of *Cyp2e1, Caspase3*, and α*Sma* were statistically normalized (Figure 2F).

Regarding the NAE-PPARα signaling system, our results indicated decreased gene expression levels of *Ppar*α and *Faah*, and an increased gene expression of *Nape-pld* in the liver of mice sacrificed 6 h after treatment (^*^P < 0.05) (Figure 2G). However, after 24 and 48 h of treatment, we observed an increase in the gene expression levels of *Nape-pld* and *Faah* (^*/***^P < 0.05/0.001). No changes were observed in the liver levels of AEA and DHEA after APAP administration (Figure 2H). In contrast, the liver levels of PEA and OEA were elevated after 24 h of APAP treatment and then normalized after 48 h (Figure 2H).

### Effect of acute APAP on the circulating levels of cholesterol, triglycerides, and hepatic injury-related enzymes in a time-dependent manner

We observed increases in the plasma levels of LDL after 6 and 24 h, triglycerides, VLDL and ALP after 24 and 48 h, AST after 6, 24, and 48 h, and ALT after 24 h of acute APAP administration (^*/**/***^P < 0.05/0.01/0.001; Table 3). In contrast, the plasma levels of HDL were decreased after 24 and 48 h of administration. No significant differences in the plasma levels of total cholesterol and γGT were found (Table 3).

**Table 3:** Effects of acute APAP (750 mg/kg) on the circulating levels of triglycerides, cholesterol and hepatic injury-related enzymes^†^.

	**Vehicle**	**APAPx1 6h**	**APAPx1 24 h**	**APAPx1 48 h**
Triglycerides (mg/dL)	73.14 ± 7.88	97.50 ± 18.11	136.20 ± 12.91^**^	129.80 ± 12.99^**^
Total cholesterol (mg/dL)	105.40 ± 3.23	109.83 ± 9.37	130.20 ± 13.81	95 ± 8.17
HDL (mg/dL)	59.24 ± 2.55	50.00 ± 10.24	52.39 ± 6.77^*^	41.60 ± 7.87^*^
LDL (mg/dL)	24.63 ± 3.78	40.33 ± 4.34^*^	43.47 ± 6.13^*^	32.40 ± 5.89
VLDL (mg/dL)	17.17 ± 3.65	19.50 ± 3.62	27.24 ± 2.58^*^	25.96 ± 2.60^*^
ALP (U/L)	22.80 ± 1.78	53.67 ± 14.75	67.39 ± 5.77^***^	73.58 ± 6.05^***^
AST (U/L)	61.25 ± 2.03	144.00 ± 33.78^*^	112.11 ± 11.37^**^	85.78 ± 5.40^**^
ALT (U/L)	36.20 ± 7.12	47.80 ± 5.47	131.60 ± 33.91^*^	49.33 ± 5.14
γGT (U/L)	5.45 ± 0.75	8.63 ± 2.20	6.72 ± 0.33	6.72 ± 0.74

† Data are expressed as mean ± S.E.M. Bonferroni test:

*/**/*** *P < 0.05/0.01/0.001 vs. vehicle-treated group*.

### Effect of repeated APAP and resting on hepatic injury-related factors and the NAE-PPARα signaling system in the mouse liver

**Figure 3:**
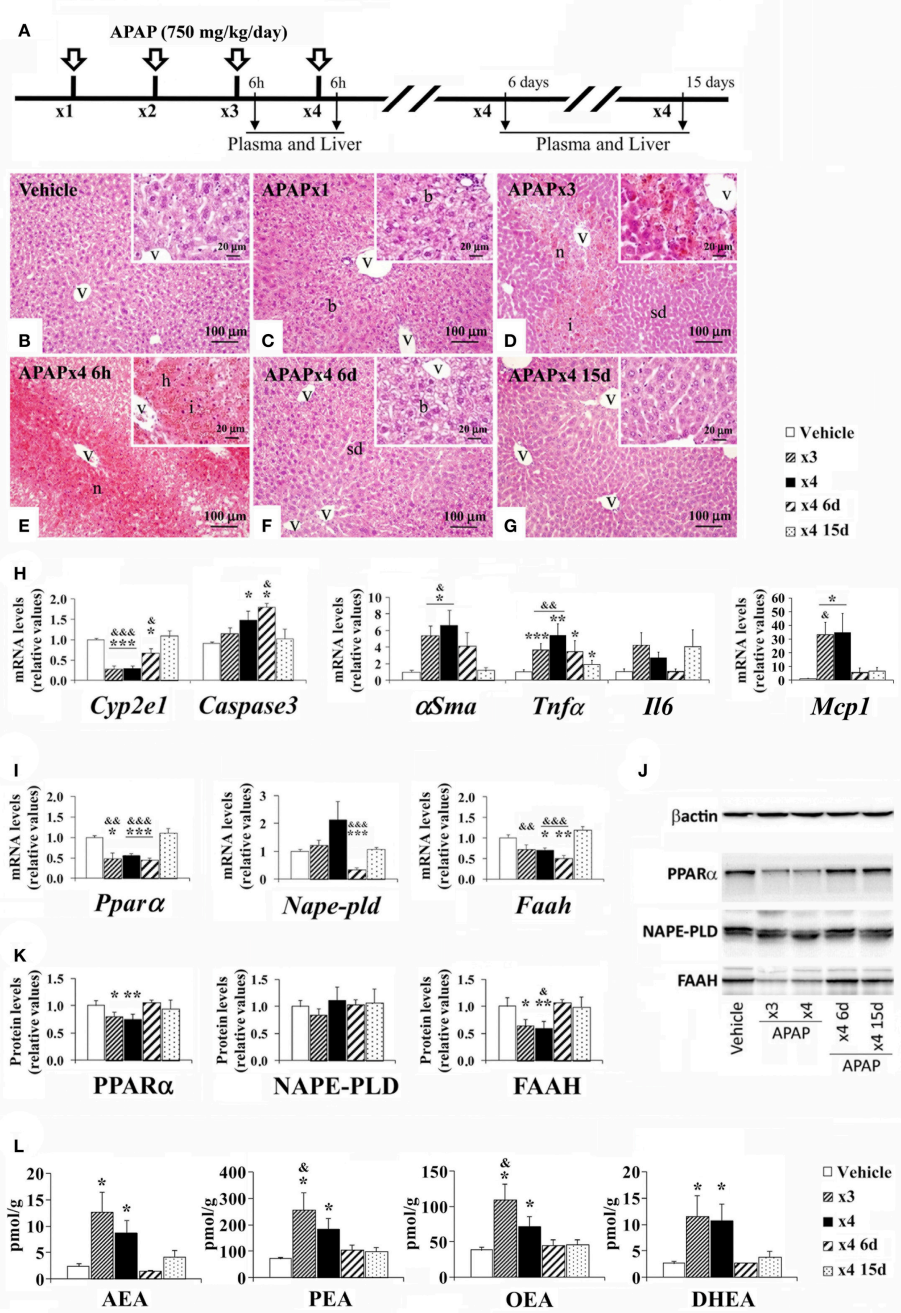
Effects of repeated APAP administration (750 mg/kg/day) on histophatology, the gene expression levels of hepatic injury-related factors, the gene/protein expression levels of NAE/PPARα-related components, and the NAE levels in the mouse liver after 6 h, 6, and 15 days from treatment. **(A)** Timeline of the experimental design. **(B–G)** Representative photomicrographs showing low and high (insets)—magnification views of H-E-stained liver sections. b, ballooning; h, hemorrhage; i, inflammation; sd, sinusoidal dilatation; v, blood vessel. **(H**) Quantification of mRNA levels of *Cyp2e1, Caspase3*, α*Sma, Tnf*α, *Il6*, and *Mcp1*. **(I–K**) Quantification of mRNA and protein levels of *Ppar*α/PPARα, *Nape-pld*/NAPE-PLD and *Faah*/FAAH. **(J)** Representative immunoblots. **(L)** Quantification of the liver levels of AEA, PEA, OEA, and DHEA. Histograms represent the mean ± S.E.M. (*n* = 6). Bonferroni test: ^*/**/***^P < 0.05/0.01/0.001 vs. vehicle-treated group; ^&/&&/&&&^*P* < 0.05/0.01/0.001 vs. APAPx4 15 days group.

Mice were treated with repeated overdoses of APAP (750 mg/kg/day) for 3 and 4 days and then sacrificed 6 h, 6 days, and 15 days (recovery) after the last oral administration (Figure 3A). We observed effects of repeated APAP and resting on the histopathology in the mouse liver. Repeated APAP administration augmented liver injury as a destruction of liver parenchyma architecture (sinusoidal dilatation and ballooning), extensive pericentral hepatic necrosis, hemorrhage and inflammation (lymphocytes infiltration) were found after increasing the number of doses (Figures 3B–G). Thus, sinusoidal dilatation and ballooning were found in the hepatic parenchyma of acute APAP-treated mice (Figure 3C). Moderate necrosis and mild sinusoidal dilatation in the hepatic parenchyma of 3-day APAP-treated mice were observed (Figure 3D). Extensive necrosis, infiltration and hemorrhage characterized the hepatic parenchyma of the 4-day APAP-treated mice (Figure 3E). When the repeated APAP administration was suspended for 6 days, the histology of the hepatic parenchyma was significantly ameliorated as minimal sinusoidal dilatation and ballooning were observed (Figure 3F). Histopathological changes were dramatically suppressed after 15 days without APAP administration (Figure 3G). Regarding the hepatic damage-related factors (Figure 3H), our results indicated a decreased gene expression of *Cyp2e1* and increased gene expressions of α*Sma, Tnf*α, and *Mcp1* in the liver of mice sacrificed 6 h after a 3-day and 4-day treatment of APAP (^*/**/***^P < 0.05/0.01/0.001). Interestingly, an increased gene expression of *Caspase3* was also detected in the liver of mice with the 4-day treatment of APAP and in the liver of mice sacrificed 6 days after the last administration of the 4-day treatment (^*^P < 0.05) (Figure 3H). Only after a resting period of 15 days from the last administration of the 4-day APAP treatment did we observe a normalization of the gene expression of these hepatic damage-related factors (^&/&&/&&&^*P* < 0.05/0.01/0.001) (Figure 3H).

Regarding the PPARα signaling machinery (Figures 3I–K), our results indicated a decreased gene and protein expression of *Ppar*α*/*PPARα and *Faah/*FAAH in the liver of mice sacrificed 6 h after a 4-day treatment of APAP (^*/**/***^P < 0.05/0.01/0.001). Interestingly, decreased gene expression levels of *Ppar*α, *Nape-pld* and *Faah* were also observed in the liver of mice sacrificed 6 days after a 4-day treatment (^**/***^P < 0.01/0.001) (Figure 3I). The significance of the changes observed in the gene and protein expression of *Ppar*α*/*PPARα and *Faah/*FAAH were all abolished after a resting period of 15 days (Figures 3I,K). No changes were observed in the protein expression of NAPE-PLD after the repeated APAP administration or resting (Figure 3K). Our results also indicated that the liver levels of AEA, PEA, OEA and DHEA were elevated in the mice treated with APAP for 3 and 4 days (^*^P < 0.05) (Figure 3L). The significance of the changes observed in the liver levels of NAEs was abolished after a resting period of 6 and 15 days.

### Effect of repeated APAP and resting on the circulating levels of cholesterol, triglycerides, and hepatic injury-related enzymes

The plasma levels of LDL, VLDL and the hepatic enzymes AST, ALT and γGT were elevated in mice treated with APAP for 4 days (^*/**/***^P < 0.05/0.01/0.001) (Table 4). After a resting period of 6 days from the fourth administration of APAP, the plasma levels of ALP, AST and γGT were also elevated. However, a resting period of 15 days normalized the plasma levels of LDL, ALP, AST, ALT and γGT (^&&/&&&^*P* < 0.01/0.001). No significant differences in the plasma levels of triglycerides, total cholesterol and HDL were found (Table 4).

**Table 4:** Effects of repeated APAP (750 mg/kg/day) and resting for 6 and 15 days on the circulating levels of triglycerides, cholesterol and hepatic injury-related enzymes^†^.

	**Vehicle**	**APAPx3**	**APAPx4**	**APAPx4 6 days**	**APAPx4 15 days**
Triglycerides (mg/dL)	85.83 ± 18.24	131.66 ± 20.47	102.50 ± 15.18	76.33 ± 5.00	87.33 ± 9.82
Total cholesterol (mg/dL)	102.50 ± 4.30	118.67 ± 13.66	103.33 ± 13.59	104.22 ± 10.63	100.17 ± 5.24
HDL (mg/dL)	60.70 ± 3.01	58.67 ± 10.81	48.40 ± 5.90	51.43 ± 2.56	59.83 ± 4.98
LDL (mg/dL)	27.22 ± 2.19	33.67 ± 9.26	49.47 ± 3.14^***^^/^^###^^/^^&&^	19.87 ± 2.07	22.87 ± 6.15
VLDL (mg/dL)	14.63 ± 1.58	26.33 ± 4.09^*^	20.83 ± 2.79^*^	15.27 ± 1.00	17.47 ± 1.96
ALP (U/L)	24.17 ± 2.32	28.00 ± 3.64	36.83 ± 10.20	98.53 ± 6.22^***^^/^^&&&^	31.67 ± 7.41
AST (U/L)	62.44 ± 2.29	208.40 ± 57.43^*^	318.67 ± 33.28^**^^/^^&&^	90.67 ± 10.63^*^	102.75 ± 7.83^*^
ALT (U/L)	30.24 ± 3.19	61.67 ± 4.51^***^	308.50 ± 43.24^**^^/^^&&^	34.78 ± 1.71	59.88 ± 16.42
γGT (U/L)	4.89 ± 0.5	9.78 ± 2.82	18.70 ± 1.40^***^^/^^&&&^	9.57 ± 1.12^**^	6.90 ± 1.15

† Data are expressed as mean ± S.E.M. Bonferroni test:

*/**/*** P < 0.05/0.01/0.001 vs. vehicle-treated group;

### P < 0.001 vs. APAPx4 6 days group;

&&/&&& *P < 0.01/0.001 vs. APAPx4 15 days group*.

### Effect of repeated APAP on hepatic injury-related factors and the circulating levels of cholesterol, triglycerides, and hepatic injury-related enzymes in the *Pparα*^−/−^ mouse liver

**Figure 4:**
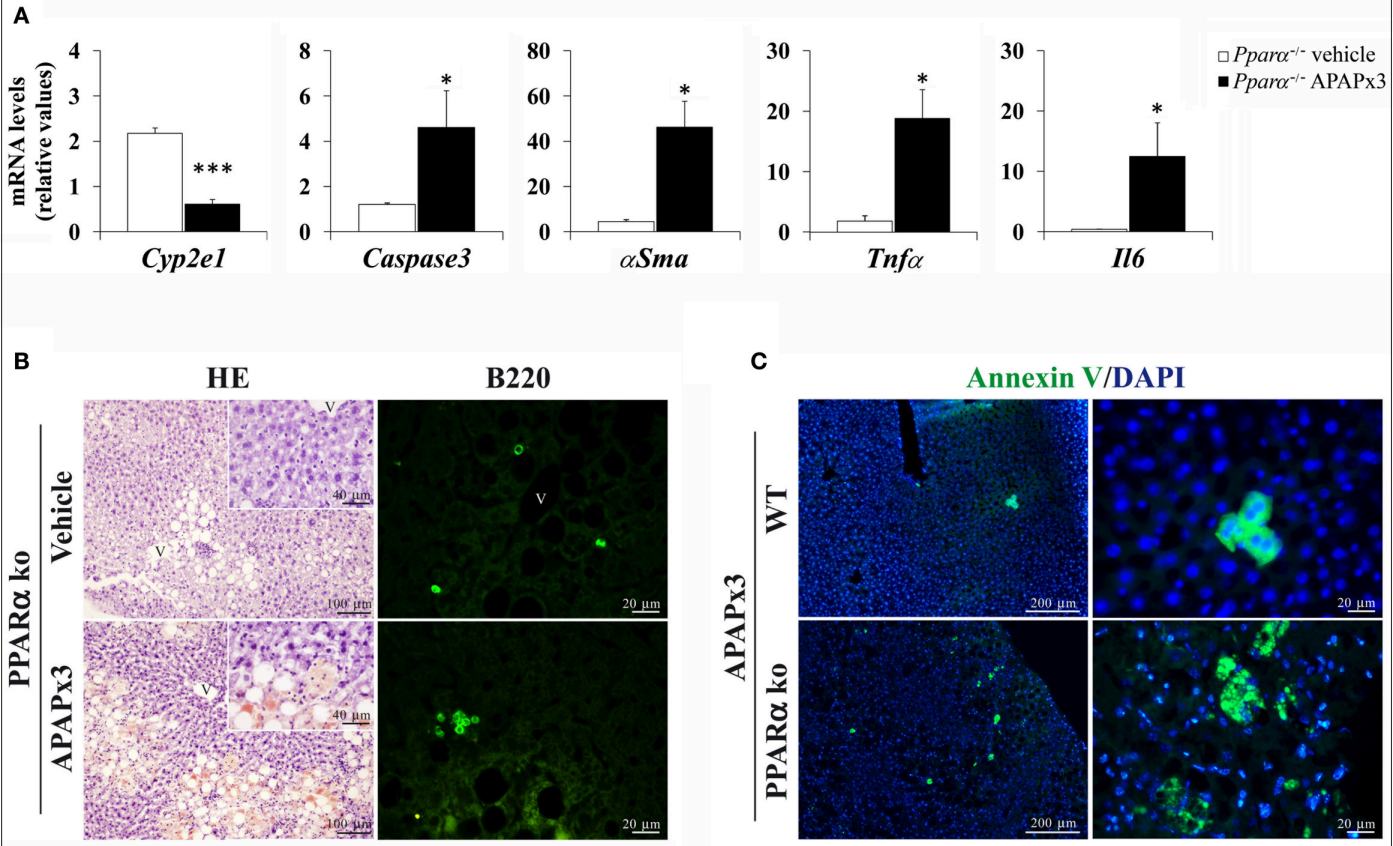
Effects of repeated APAP administration (750 mg/kg/day) on the expression of hepatic injury-related factors in the liver of *Ppar*α^−/−^ mice. **(A)** Quantification of mRNA levels of *Cyp2e1, Caspase3*, α*Sma, Tnf*α, and *Il6*. Histograms represent the mean ± S.E.M. (*n* = 6). Student's *t* test: ^*/***^P < 0.05/0.001 vs. vehicle-treated *Ppar*α^−/−^ group. **(B)** Representative photomicrographs showing low and high (insets)—magnification views of H-E staining and B220 (CD45R) immunofluorescence in liver sections. **(C)** Representative photomicrographs showing fluorescent labeling for the apoptotic Annexin V and DAPI in the liver of CD1 and *Ppar*α^−/−^ mice repeatedly treated with APAP. V, blood vessel.

As observed previously, the results also indicated a decreased gene expression of *Cyp2e1*, as well as an increase in the gene expression levels of *Caspase3*, α*Sma, Tnf*α and *Il6* in the liver of *Ppar*α^−/−^ mice sacrificed 6 h after a 3-day treatment of APAP (^*/***^P < 0.05/0.001) (Figure 4A).

In the hepatic parenchyma of *Ppar*α^−/−^ mice, the histopathology was compatible with an increase in hepatic fat accumulation and T lymphocyte infiltration associated with B220 (CD45) labeling after repeated APAP administration (Figure 4B). Interestingly, the apoptotic Annexin V marker was highly detected in the hepatocytes of *Ppar*α^−/−^ mice than those of CD1 mice when they were repeatedly exposed to APAP (Figure 4C).

The plasma levels of triglycerides, VLDL and alkaline phosphatase (ALP) were elevated in *Ppar*α^−/−^ mice treated with APAP for 3 days (^**^P < 0.01) (Table 5).

**Table 5:** Effects of repeated APAP (750 mg/kg/day) on the circulating levels of triglycerides, cholesterol and hepatic injury-related enzymes in *Ppar*α^−/−^ mice^†^.

	***Pparα*^−/−^vehicle**	***Pparα*^−/−^APAPx3**
Triglycerides (mg/dL)	86.75 ± 3.41	192.80 ± 20.17^**^
Total cholesterol (mg/dL)	101.00 ± 3.94	147.40 ± 45.46
HDL (mg/dL)	60.25 ± 4.53	68.60 ± 22.55
LDL (mg/dL)	23.40 ± 3.30	51.45 ± 21.76
VLDL (mg/dL)	17.35 ± 0.68	38.56 ± 4.03^**^
ALP (U/L)	8.25 ± 1.66	16.20 ± 2.82^**^
AST (U/L)	170.75 ± 38.89	78.00 ± 49.26
ALT (U/L)	44.75 ± 12.12	110.8 ± 70.99
γGT (U/L)	4.88 ± 1.73	4.28 ± 1.59

† Data are expressed as mean ± S.E.M. Bonferroni test:

** *P < 0.01 vs. vehicle-treated Pparα^−/−^ group*.

### Correlation between NAE levels and hepatic damage-related factors

To assess the relevance of NAE changes in apoptosis and inflammation, we statistically analyzed the correlation between the liver levels of NAEs and the liver gene expression levels of hepatic damage-related factors (*Casp3*, α*Sma, Mcp1, Tnf*α, and *Il6*). The results indicated that the liver levels of AEA, PEA and DHEA, but not OEA, positively correlated with *Mcp1* gene expression (Table 6), whereas only DHEA positively correlated with the gene expression levels of α*Sma, Mcp1*, and *Tnf*α, but not *Casp3* and *IL6* (Table 7). These results indicated that there is an inflammatory role of the NAE signaling in APAP-induced liver injury.

**Table 6:** Statistical correlation between NAEs levels and *Mcp-1* gene expression in liver^†^.

	**AEA**		**PEA**		**OEA**		**DHEA**	
	***r***	***p***	***r***	***p***	***r***	***p***	***r***	***p***
*Mcp1*	0.8969	**<0.01**	0.7875	**<0.05**	0.5730	0.1787	0.9658	**<0.001**

† *Pearson correlation coefficients (r) and P values are indicated*.

*Bold values indicate the statistical significance of the P values by Pearson correlation*.

**Table 7:** Statistical correlation between the gene expression levels of hepatic damage-related factors and DHEA levels in liver^†^.

	***Casp3***		***αSma***		***Mcp1***		***Tnfα***		***Il6***	
	***r***	***p***	***r***	***p***	***r***	***p***	***r***	***p***	***r***	***P***
DHEA	−0.122	0.7943	0.7944	**<0.05**	0.9658	**<0.001**	0.7760	**<0.05**	0.2710	0.5567

† *Pearson correlation coefficients (r) and P values are indicated*.

*Bold values indicate the statistical significance of the P values by Pearson correlation*.

### Repeated APAP in mouse and APAP overdose in human decreased the immunohistochemical expression of PPARα and FAAH in liver

**Figure 5:**
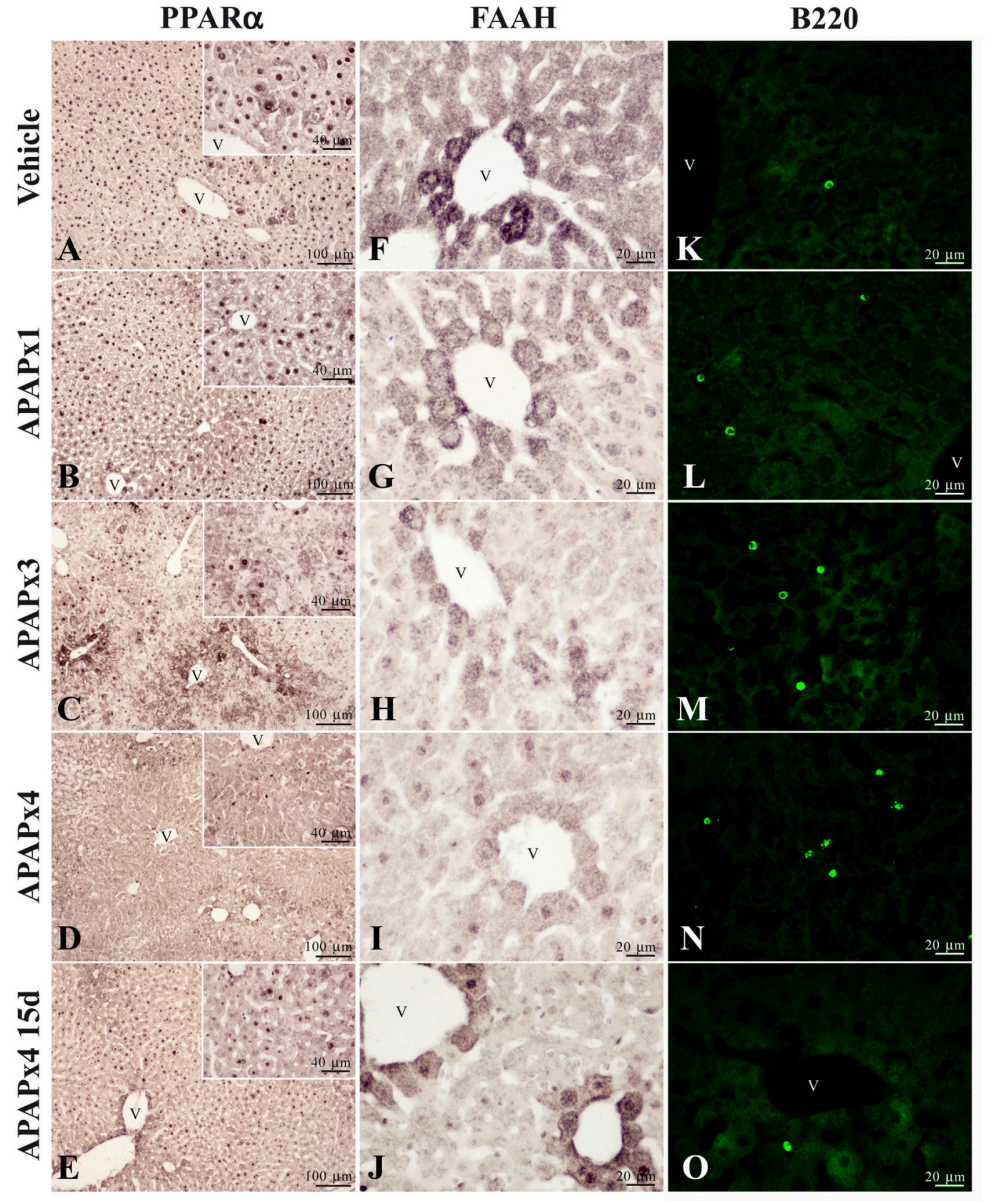
Representative high-magnification photomicrographs showing the immunohistochemical expression of PPARα **(A–E)** and FAAH **(F–J)**, and the immunofluorescence expression of B220 (CD45R) **(K–O)**, in liver sections of mice treated with acute and repeated oral overdoses of APAP after 6 h and 15 days from treatment. v, blood vessel.

**Figure 6:**
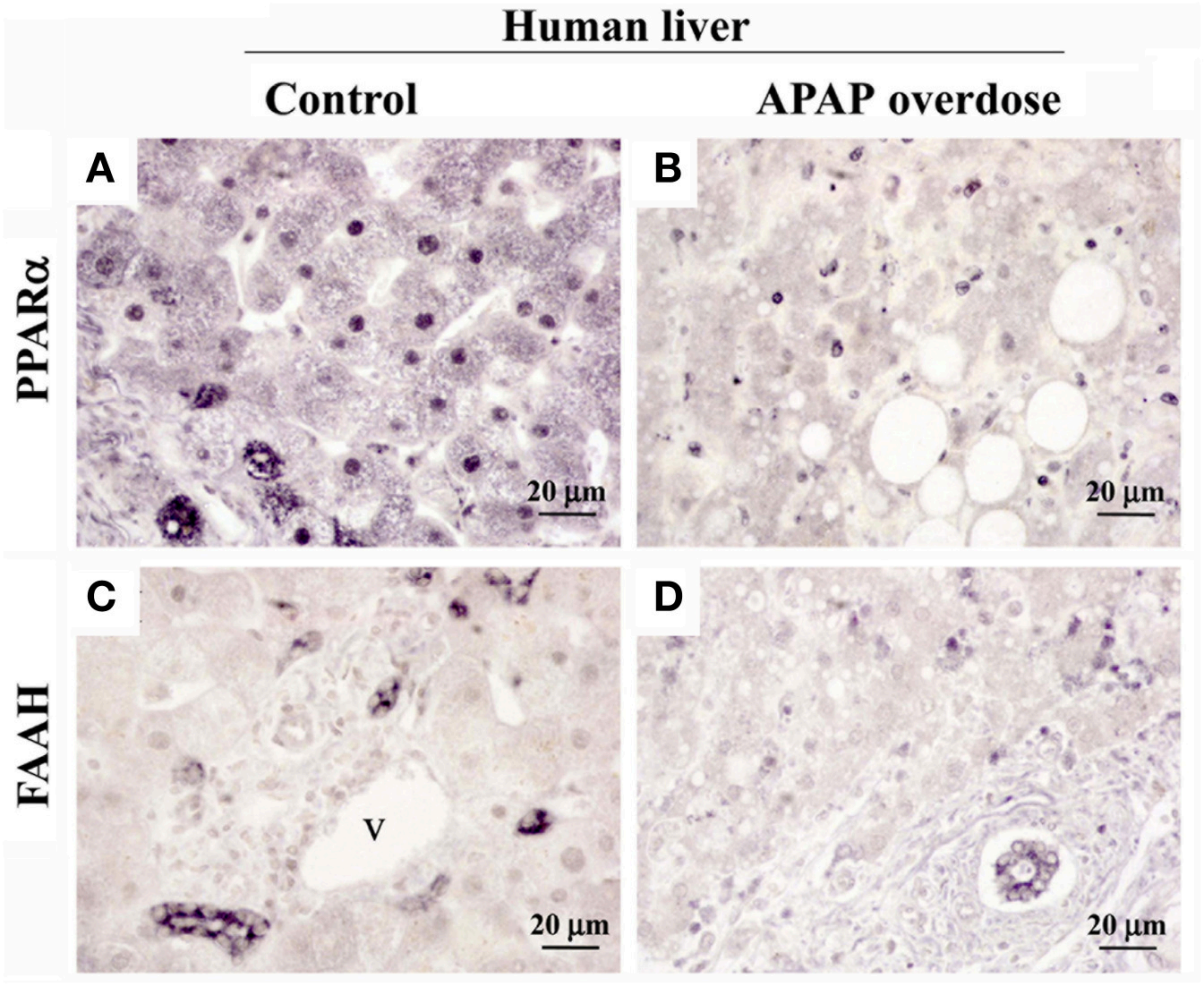
Representative high-magnification photomicrographs showing the immunohistochemical expression of PPARα **(A,B)** and FAAH **(C,D)** in liver sections of a patient with an acute hepatic failure caused by a prolonged consumption of paracetamol. v, blood vessel.

A reduced immunostaining of PPARα (Figures 5A–E) and FAAH (Figures 5F–J) in the liver of mice treated with APAP supported the results previously obtained from the gene and protein expression analysis. We also observed an increase in the infiltration of T lymphocytes expressing B220 (Figures 5K–O) in mice repeatedly exposed to APAP. T lymphocyte infiltration was almost completely abolished after a resting period of 15 days. Immunohistochemical analysis in the human liver after APAP overdose also confirmed the PPARα and FAAH decreases (Figure 6). PPARα immunoreactivity was not only detected as a nuclear labeling in most hepatocytes and stellate cells but also detected in the cytoplasm of some of them (Figures 5A, 6A). FAAH labeling was only detected in the cytoplasm of hepatocytes, especially in those hepatocytes surrounding the blood vessels (Figures 5F, 6C).

## Discussion

**Figure 7:**
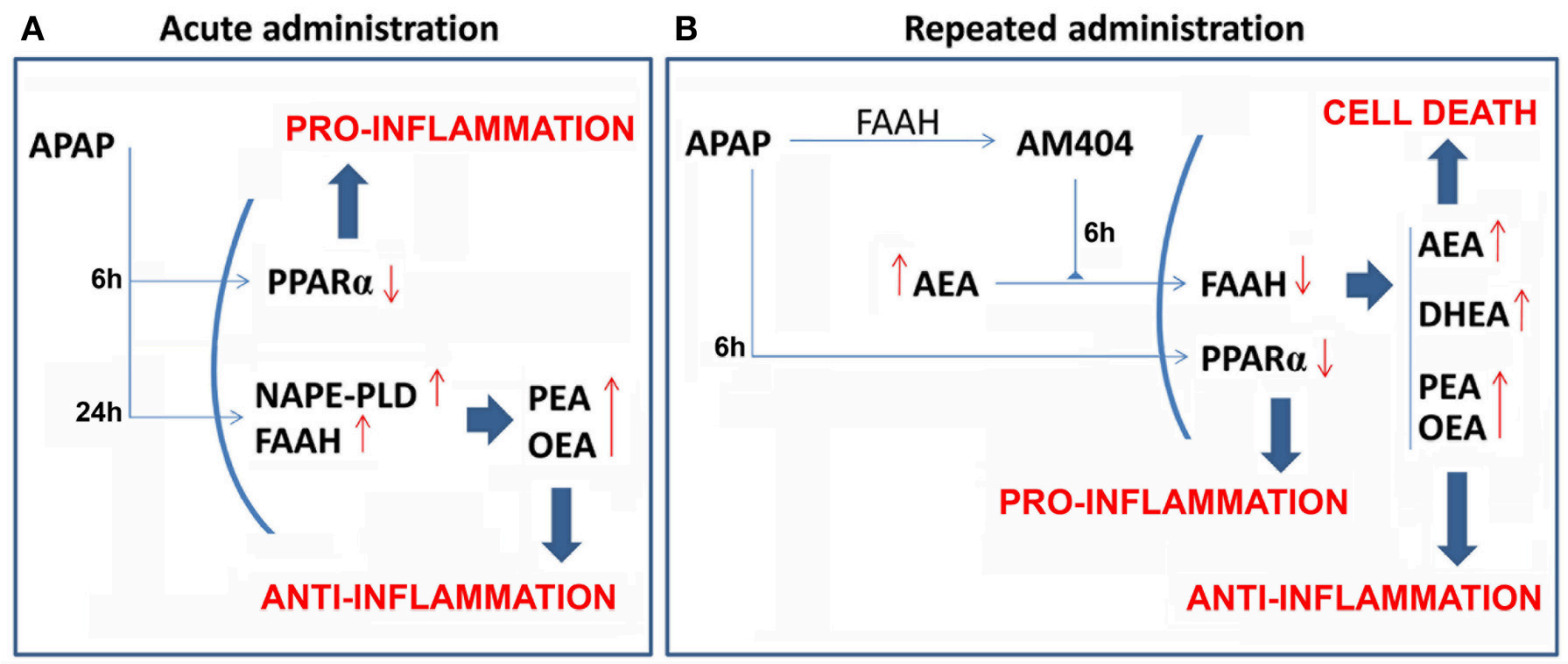
Scheme that summarizes the acute **(A)** and repeated **(B)** effects of APAP on the liver expression of the NAE/PPARα-related machinery involved in the signaling (PPARα), synthesis (NAPE-PLD) and degradation (FAAH) of the endogenous NAE derivatives AEA, PEA, DHEA, and OEA. Red arrows indicate the increased/decreased expression/levels.

The present study demonstrated that the oral overdose of acetaminophen, which was acutely and repeatedly administered to induce liver injury, modified the liver expressions of the NAE-based anti-inflammatory system composed by the PPARα, the NAEs synthesis enzyme NAPE-PLD and the NAEs degradation enzyme FAAH. 6 h after treatment, acute and repeated administrations of APAP induced a decreased expression of PPARα, which was observed in the hepatic HepG2 cells and in the mouse liver (see Figure 7 for a summary). This effect on PPARα expression was completely reversed after 24 h from acute APAP administration and after 6 days from the last dose of the repeated APAP administration. Moreover, FAAH expression was decreased in the mouse liver after 6 h from acute and repeated APAP treatment and was restored or even up-regulated after 24 h from acute administration and after 15 days from the last dose of the repeated APAP administration. In contrast, *Nape-pld* gene expression was increased after 24 h of APAP incubation in the hepatic HepG2 cells and after 6, 24, and 48 h from acute APAP treatment in the mouse liver. Repeated administration of APAP for 4 days also induced a tendency in the increased expression of NAPE-PLD, which was reversed or even down-regulated after 6 and 15 days from the last APAP administration. As expected, the changes in the liver expression of these NAE metabolic enzymes result in altered levels of the NAEs AEA, OEA, PEA, and DHEA in the liver. The increased levels of OEA and PEA, but not AEA and DHEA, can be explained by the increased gene expression of *Nape-pld* (3-fold increase) in the mouse liver after 24 h of APAP administration. The acute APAP effect on *Nape-pld* gene expression and OEA levels in the mouse liver was also observed in the hepatic HepG2 cells after 24 h of incubation. Regarding the livers of mice treated with repeated APAP administration, the increase in the levels of AEA, PEA, OEA, and DHEA agree with the outstanding lower gene/protein expression of FAAH. However, we cannot ignore the increased gene expression of FAAH after acute APAP administration in HepG2 cells and the mouse liver. The increase in NAE levels observed in our study can be interpreted considering a higher influence of acute APAP administration on NAPE-PLD activity over FAAH, which contrasts the main effect of repeated APAP administration in FAAH over NAPE-PLD expression. Immunohistochemistry in the human liver with hepatoxicity caused by abusive APAP consumption confirmed the decreased expressions of PPARα and FAAH previously observed in our animal models regarding a time-course of 6 h from the last APAP administration (acute or repeated). These results indicate that the actions of APAP on the NAEs-PPARα signaling system are preserved through species, which suggests an important role in the response to the toxic action of this drug and opens interesting possibilities for investigating these dynamic changes in NAEs machinery and signaling on diagnosis, prognosis and treatment of APAP-induced liver injury.

Because of the well-known role of PPARα in the regulation of metabolic function, liver regeneration and fibrosis induction, it is possible that the APAP-induced recruitment of the NAE-based anti-inflammatory signaling system may contribute to the pathogenesis and final outcomes of AILI. In the present study, we found concordance when we compared the expression of the NAE metabolic machinery over time, the expression of the hepatic damage-related factors and the histopathological changes in the liver. In acute and repeated APAP administration for 4 days, we found that severe histopathological changes, which include sinusoidal dilatation, ballooning, lymphocyte infiltration (inflammation), hemorrhage and extensive pericentral hepatic necrosis, were associated with impairment in hepatic damage-related factors, such as a decrease in gene expression of the xenobiotic metabolism factor *Cyp2e1* and increases in gene expression levels of the proapoptotic factor *Caspase3*, the fibrogenic factor α*Sma*, the pro-inflammatory cytokines *Tnf*α and *Il6*, and the monocyte/macrophage migration/infiltration factor *Mcp1* (also known as CCL2). Moreover, the greatest damage in mouse liver (acute and repeated administration of APAP for 4 days) was accompanied by decreased expressions of PPARα and FAAH, and by a positive correlation between *Mcp1* and NAE levels. To further assess the role of PPARα in hepatic damage caused by APAP, we evaluated the effects of the repeated administration of APAP on the liver of *Ppar*α^−/−^ mice. In these mice, we observed an up to four-fold increase in the gene expression of *Caspase3* and an up to 10-fold increase in the gene expression levels of α*Sma, Tnf*α, and *Il6*. Impairment inflammation in *Ppar*α^−/−^ mouse liver was confirmed after the qualitative increase in the number of cells expressing the naive T lymphocyte marker B220 (CD45R) and the number of hepatocytes expressing the apoptotic Annexin V. These results indicate a higher liver vulnerability to APAP in the absence of PPARα. Previous studies described that the lack of CYP2E1-metabolic activation and oxidative stress following APAP treatment caused an increase in mitochondrial ROS formation (Jiang et al., 2015) and a suppression of PPARα-regulated pathways on fatty acid oxidation (Chen et al., 2009). Our results are in strong agreement with a previous report demonstrating that elevated reactive oxygen species generated during APAP-induced hepatotoxicity are partially mediated by reduced PPARα activity linked to the lack of UCP2 induction (Patterson et al., 2012). Regarding this information, the down-expression of *PPAR*α after 6 h of APAP incubation in the hepatic HepG2 cells is consistent with a context of cytochrome P450-oxidase system deprivation, as the hepatic HepG2 cell line has low expression of CYP2E1, among other cytochrome P450 isozymes (Van Summeren et al., 2011). These results framed in a 6-h-time course were confirmed in the mouse models of acute and repeated treatment as decreased expression levels of *Cyp2E1*, PPARα, and FAAH can be associated. Thus, alteration of CYP2E1 activity could also be necessary in order to find an effect of APAP on FAAH expression and NAEs levels in the liver. Additionally, we cannot disregard that the over-expression of the NAEs-PPARα signaling system in response to a 24-h APAP hepatotoxicity in the hepatic HepG2 cells could be produced in a P450-oxidase system-independent manner.

Acute and repeated APAP administration resulted in a PPARα down-expression and likely explains the lack of anti-inflammatory properties that characterize APAP actions (Abdelmegeed et al., 2011; Ratziu et al., 2016). In agreement with the increased gene expression of the pro-inflammatory cytokine *Tnf*α after repeated APAP overdose, PPARα activity has been described to be closely down-regulated by pro-inflammatory cytokines (Straus and Glass, 2007). In this regard, few data are present in the literature about the immune response of PPARα in the secretion of MCP1 (Antonelli et al., 2012). Our results also indicated that FAAH down-expression and the lack of AEA degradation after APAP treatment could be implicated in the hepatotoxic mechanism of APAP that includes elevated levels of LDL, VLDL, and hepatic transaminases in plasma, altered gene expression of hepatic damage-related factors of fibrosis, inflammation and necrosis. The elevated gene expression levels of the hepatic fibrosis marker α*Sma* and the pro-apoptosis marker *Caspase3* in the liver of mice with repeated APAP administration indicates that the liver fibrosis is likely promoted by hepatocyte cell death (Siegmund et al., 2013). A previous report demonstrated higher levels of AEA in the plasma of severe hepatitis and cirrhosis patients (Biswas et al., 2003). These authors also demonstrated that AEA-induced cell death in human HepG2 cells and primary hepatocytes was preceded by the activation of pro-apoptotic signaling. Focusing on the livers of the repeated APAP-treated mice, our results also agree with the fact that the elevated levels of AEA-induced hepatocyte cell death (*Caspase3*) were determined by the reduced expression of FAAH (Siegmund et al., 2006). In contrast to the hepatic stellate cells, the high levels of FAAH in hepatocytes may confer less sensitivity to AEA and more resistance to pro-fibrotic signals (α*Sma*). Different mechanisms of AEA-induced cell death have been associated with CB1 and CB2 stimulation, ceramide generation, JNK activation, ROS generation, and caspase3 activation and apoptosis (Siegmund and Schwabe, 2008). DHEA, which is produced by the same pathway as AEA, could also be a local anti-inflammatory mediator, as it is able to prevent the formation of platelet-leukocyte aggregates and ameliorate inflammation-associated degenerative conditions (Yang et al., 2011; Park et al., 2016).

Several studies have also reported that PPARα activation attenuates liver fibrosis (Ip et al., 2004). The circulating levels of the PPARα agonists OEA and PEA were significantly higher in cirrhotic patients (Caraceni et al., 2010). Moreover, OEA was described to suppress the pro-fibrotic cytokine TGF-β1 signaling in mouse models of hepatic fibrosis, an improvement that was lost in PPARα knockout mice (Chen et al., 2015). In agreement with previous studies, our results indicated that the increased expression of the fibrosis marker α*Sma* in acute and repeated AILI was accompanied by a decreased expression of PPARα, a fact that contrasted the high concentration of OEA. APAP in PPARα deficiency increased the gene expression of α*Sma* and confirmed PPARα-related molecular mechanisms mediating APAP-induced liver injury.

Previous studies showed that the analgesic and hypothermic actions of APAP are partially mediated through the NAE-degrading enzyme FAAH, which facilitates the hydrolysis of APAP into the potent TRPV1 agonist AM404 (De Petrocellis et al., 2000; Högestatt et al., 2005; Ghanem et al., 2016). AM404 inhibits the carrier-mediated transport of AEA resulting, in turn, in a reduction of FAAH activity (Beltramo et al., 1997; Howlett, 2002; Ayoub et al., 2011). As expected, the repeated administration of APAP at doses of 750 mg/kg/day induced a decreased expression of FAAH and increased levels of AEA in the mouse liver. However, the acute administration of APAP in human HepG2 cells and mice did not induce clear modifications in the levels of AEA. This apparent contradiction compared to the elevated levels of OEA, DHEA, or PEA could be explained regarding the higher reactivity of the NAE-degrading enzyme FAAH with AEA than OEA and PEA as was previously demonstrated (Deutsch et al., 2002). This explanation agreed with the lack of hypothermic actions mediated by AEA in mice treated with AM404, a metabolite resulting from APAP deacetylation (Ayoub et al., 2011). There is also evidence of pharmacological interaction between APAP and PEA, whose mechanisms of action confer an enhanced effect on antihyperalgesia (Déciga-Campos and Ortíz-Andrade, 2015).

AEA and OEA have opposed pharmacological profiles, as was demonstrated in feeding behavior (Gómez et al., 2002). In the present study, both OEA and AEA are elevated after APAP and may exhibit opposed roles in the onset of fibrogenesis. OEA administration has been found to attenuate toxic (thioacetamide-induced) liver fibrosis by targeting hepatic stellate cells. It is reasonable to think that the lowering of PPARα at the initial levels can reduce the OEA-dependent protective response against AEA-induced fibrogenesis, a detrimental situation that is reversed over time. The upregulation of the system is coincident with liver recovery, to which the extent of APAP-induced deficits in PPAR signaling responsible for the acute toxic effects should be determined (Chen et al., 2015).

In conclusion, our study provides compelling evidence that the liver expression of the NAE/PPARα-based anti-inflammatory system is involved in the acute and repeated APAP overdose-induced liver injury over time. The increased hepatic content of NAEs is likely explained by changes in the liver expressions of their metabolic enzymes. The increased levels of NAEs through FAAH down-expression after repeated APAP overdose are likely associated with hepatotoxicity raised by fibrotic (α*Sma*), apoptotic (*Casp3*) and inflammatory (*Tnf*α, *Mcp1*) signaling. Moreover, the onset of pro-inflammatory events that characterize the pharmacological actions of APAP is likely explained by the decreased expression of PPARα and confirmed in the liver of *Ppar*α^−/−^ mice. Interestingly, the normalization of NAE-PPARα signaling after a resting period of 15 days supported the liver capacity for regeneration and recovery. These results may provide new pharmacological strategies for the prevention of the side effects derived from APAP-induced liver injury in humans, allowing the specific targeting of NAE-signaling elements such as PPARα and FAAH.

## Author contributions

PR, FdF, and JS were responsible for concept and design of the study. PR, AP, SA, JD, AV, LS, FP, AS, DB, AB, and RdT contributed to data acquisition. PR, EB, ML, FdF, and JS contributed to data analysis and interpretation. PR and JS drafted the manuscript. All authors were involved in revising the manuscript and approved the final version to be published.

### Conflict of interest statement

The authors declare that the research was conducted in the absence of any commercial or financial relationships that could be construed as a potential conflict of interest.

## Abbreviations

α*Sma/Acta2*: alpha smooth muscle actin
AEA: anandamide
ALT: alanine aminotransferase
AM404: N-(4-hydroxyphenyl)-arachidonylamide
AILI: APAP-induced liver injury
ALP: phosptase alkaline
APAP: acetaminophen or paracetamol
AST: aspartate aminotransferase
B220 (CD45): leukocyte common antigen
*Cyp2e1*: cytochrome P450 2E1
DAPI: 4′,6-diamidino-2-phenylindole
DHEA: docosahexaenoyl ethanolamide
DILI: drug-induced liver injury
*Faah*: fatty acid amide hydrolase
γGT: gamma-glutamyl transpeptidase
HDL: high-density lipoprotein-cholesterol
IL6: interleukin 6
LDL: low-density lipoproteins
MCP1/CCL2: monocyte chemoattractant protein-1
NAEs: N-acyl ethanolamines
*Nape-pld*: *N*-acyl phosphatidylethanolamine phospholipase D
OEA: oleoyl ethanolamide
PEA: palmitoyl ethanolamide
*Ppar*α: peroxisome proliferator-activated receptor alpha
TNFα: tumor necrosis factor α
VLDL: very low-density lipoproteins.
